# Quantifying *in vivo* collagen reorganization during immunotherapy in murine melanoma with second harmonic generation imaging

**DOI:** 10.1117/1.BIOS.1.1.015004

**Published:** 2024-05-20

**Authors:** Alexa R. Heaton, Nathaniel J. Burkard, Paul M. Sondel, Melissa C. Skala

**Affiliations:** a Morgridge Institute for Research, Madison, Wisconsin, United States; b University of Wisconsin, Department of Human Oncology, Madison, Wisconsin, United States; c University of Wisconsin, Department of Biomedical Engineering, Madison, Wisconsin, United States; d University of Wisconsin, Department of Pediatrics, Madison, Wisconsin, United States

**Keywords:** collagen, second-harmonic generation, immunotherapy, melanoma, *in vivo*, quantitative imaging

## Abstract

**Significance:**

Increased collagen linearization and deposition during tumorigenesis can impede immune cell infiltration and lead to tumor metastasis. Although melanoma is well studied in immunotherapy research, studies that quantify collagen changes during melanoma progression and treatment are lacking.

**Aim:**

We aim to image *in vivo* collagen in preclinical melanoma models during immunotherapy and quantify the collagen phenotype in treated and control mice.

**Approach:**

Second-harmonic generation imaging of collagen was performed in mouse melanoma tumors *in vivo* over a treatment time course. Animals were treated with a curative radiation and immunotherapy combination. Collagen morphology was quantified over time at an image and single-fiber level using CurveAlign and CT-FIRE software.

**Results:**

In immunotherapy-treated mice, collagen was reorganized toward a healthy phenotype, including shorter, wider, curlier collagen fibers, with modestly higher collagen density. Temporally, collagen fiber straightness and length changed late in treatment (days 9 and 12), while width and density changed early (day 6) compared with control mice. Single-fiber collagen features calculated in CT-FIRE were the most sensitive to the changes among treatment groups compared with bulk collagen features.

**Conclusions:**

Quantitative second-harmonic generation imaging can provide insight into collagen dynamics *in vivo* during immunotherapy, with key implications in improving immunotherapy response in melanoma and other cancers.

## 1 Introduction

The extracellular matrix (ECM) is a major component of the tissue microenvironment, providing a scaffold for surrounding cells and regulating cell behavior. Within the ECM, collagen is a structural protein that comprises 30% of the total protein mass in the human body and is involved in several key biological processes.^1^ In normal tissue, the ECM constantly remodels and repairs, synthesizing new collagen proteins to replace the older, degraded collagen. This process is highly regulated by a precise balance of metalloproteinases (MMPs) and MMP inhibitors.^2,3^ Within the context of cancer, homeostasis is dysfunctional, and cancer cells secrete excess amounts of MMPs, degrading the basement membrane and promoting malignant cell invasion into the interstitial matrix.^1,3^ As the tumor progresses, cancer-associated fibroblasts (CAFs) secrete excess types I and II fibrillar collagen and remodel the collagen morphology within the ECM.^4^ Often, ECM morphology reconstruction results in linearized collagen and increased stiffening within cancer tissue.^1,2^ These phenomena have been categorized as tumor-associated collagen signatures (TACSs) that summarize the collagen changes that often occur during tumorigenesis: increased deposition or density (TACS-1), more “taut” or straight fibers (TACS-2), and increased fiber alignment (TACS-3).^5,6^ These morphological changes within the tumor microenvironment affect tumor cell migration and metastasis out of the tumor while impacting immune cell recruitment and infiltration into the tumor.^1,2,5,7^

Melanoma is the deadliest of all skin cancers and accounted for 325,000 new cases with an 18% fatality rate globally in 2020.^8,9^ Current predictions expect diagnoses (+50%) and fatalities (+68%) to continue to increase over the next 17 years.^8^ This cancer type affects a wide population with metastatic disease usually driving poor outcomes. With the emergence of somewhat effective immune checkpoint blockade immunotherapy, advanced melanoma prognosis has dramatically improved over the last 25 years, from 9-month median survival rates to a substantial fraction of patients achieving durable cures.^10,11^ Immune checkpoint inhibitors have exhibited especially potent results against melanoma with the best outcomes from combination therapy versus monotherapy.^11,12^ Here, we pursue one such combination strategy, including external beam radiation therapy, immune checkpoint inhibitor, and a novel immunocytokine (IC). We have previously shown that this triple combination can cure large GD2+ melanomas in the majority of treated mice.^13-16^ Here, we continue our investigation of this murine B78 melanoma model and expand our inquiry into the tumor microenvironment with a focus on collagen.

Second-harmonic generation (SHG) imaging can be used to visualize collagen in its endogenous, label-free state. SHG is a nonlinear optical scattering phenomenon that occurs when two identical photons scatter off a noncentrosymmetric material, collagen here, producing a single photon with exactly twice the energy of the initial photons. As a result of this frequency doubling, SHG signals are always generated at half the excitation wavelength.^17-19^ SHG imaging has emerged as a valuable method to image collagen *in vitro* and *in vivo* due to its high contrast and specificity.^20-24^ There are several open source tools available to analyze and quantify collagen orientation, including CytoSpectre,^25^ an ImageJ macro TWOMBLI (The Workflow of Matrix BioLogy Informatics),^26^ ImageJ plugins FibriTool,^27^ and OrientationJ,^28^ as well as CurveAlign and CT-FIRE.^6,8,29-32^ In addition, intensity derivatives, Fourier transforms, and Hough transforms can be used to quantify fiber orientation. We chose to quantify collagen morphology from our SHG images using CurveAlign and CT-FIRE software developed by the Eliceiri lab at the University of Wisconsin. CurveAlign and CT-FIRE enabled high-throughput analysis of raw SHG images in our large dataset as well as both bulk collagen morphology parameters (coefficient of alignment and density) and single-fiber collagen morphology parameters (straightness, length, width). In addition to the CurveAlign and CT-FIRE parameters, collagen morphology also varies macroscopically between straight and curly fibers, which can be qualitatively scored for each field of view (FOV).

Although there is significant activity in melanoma immunotherapy research, we are not aware of any studies that have quantified collagen changes during melanoma treatment with immunotherapy, despite collagen comprising 70% of the skin.^33^ In addition, melanoma has been shown to be incredibly fibroblast rich, with human melanoma tumors recruiting activated CAFs.^34-37^ As CAFs are one of the key cell types implicated in pro-tumor collagen and MMP deposition, we anticipate that collagen changes within melanoma may be informative to patient prognosis and survival.^34,37-39^ Prior work has used SHG imaging to probe collagen changes primarily in breast^6,40-47^ and pancreatic cancer^48-53^ with some analyses in melanoma.^22,54,55^ We aim to expand this analysis to melanoma in the context of radiotherapy and immunotherapy treatment. We aim to characterize the melanoma collagen morphology and to test whether the melanoma collagen phenotype shifts away from tumor-associated signatures and toward a healthy tissue phenotype during combination therapy.

Here, we quantify collagen morphology features from *in vivo* SHG images of mouse melanoma tumors during a combination of radiation and immunotherapy versus treatment with a phosphate-buffered saline (PBS) vehicle. We examine collagen reorganization and phenotypic changes with temporal context and use the quantitative features from CurveAlign and CT-FIRE software to evaluate collagen changes at the FOV level and the single-fiber level.

## 2 Methods

### 2.1 Mouse Model

#### 2.1.1 Preparation of mouse tumor model

Animals were housed and treated under an animal protocol approved by the Institutional Animal Care and Use Committee at the University of Wisconsin, Madison. Genetically modified C57BL/6 mice (Jax 000664), with an mCherry reporter within their CD8 T cells, were created at the University of Wisconsin Genome Editing and Animal Models core and used for these studies. The data presented here will not discuss the mCherry expressing CD8 T cells as the focus is just on collagen changes. A separate paper, in preparation, will feature the T-cell data obtained from these radio-immunotherapy-treated mice. Mice were successfully bred and maintained by the University of Wisconsin Biomedical Research Model Services. Equal numbers of male and female mice, ages 2 to 6 months, were used in all studies. A total of 16 mice were imaged, including pretreatment mice (*n* = 4), vehicle-treated mice (*n* = 6), and treated mice (*n* = 6).

B78-D14 (B78) melanoma is a poorly immunogenic cell line derived from B78-H1 melanoma cells, which were originally derived from B16 melanoma.^56-58^ These cells were obtained from Ralph Reisfeld (Scripps Research Institute) in 2002. B78 cells were transfected with functional GD2/GD3 synthase to express the disialoganglioside GD2,^56,58^ which is overexpressed on the surface of many human tumors, including melanoma.^59^ These B78 cells were also found to lack melanin. B78 cells were grown in RPMI-1640 (Gibco, Waltham, Massachusetts, United States) supplemented with 10% FBS and 1% penicillin/streptomycin, with periodic supplementation with 400 *μ*g G418 and 500 *μ*g Hygromycin B per mL. Mycoplasma testing was performed every 6 months. B78 tumors were engrafted by shallow, intradermal flank injection of 2 × 10^6^ tumor cells. The intradermal injection was confirmed by palpating the tumors—where confirmed intradermal tumors move with the skin during skin displacement, as previously published.^15^ We have previously developed successful immunotherapy regimens for mice bearing these B78 tumors, enabling the cure of mice with measurable tumors (~100 mm^3^ volume).^13,14,16^ These cured mice have demonstrated tumor-specific T-cell mediated memory, as detected by rejection of rechallenge with the same versus immunologically distinct tumors. Here, we continue our investigation of the B78 tumor model and expand our work by investigating collagen changes that occur during therapy. Tumor size was determined using calipers and volume approximated as

$$\text{tumor volume}=\frac{\left({\text{tumor width}}^{2}\times \text{tumor length}\right)}{2}.$$

#### 2.1.2 Therapy administration

**Fig. 1:**
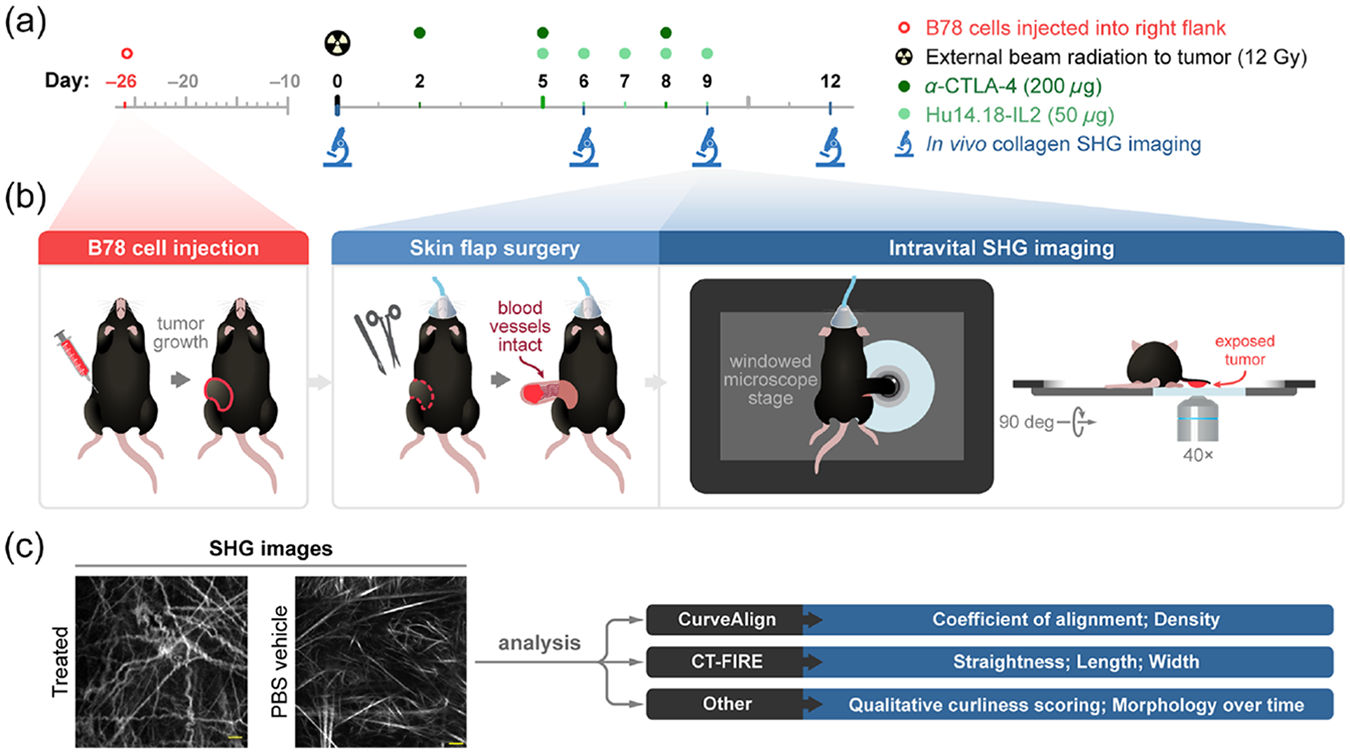
*In vivo* SHG imaging and treatment experimental workflow. (a) Experimental workflow began with intradermal inoculation of 2 × 10^6^ B78 melanoma cells into the right flank of our reporter mice. Tumors were monitored weekly until they reached ~150 mm^3^ volume (requiring ~26 days). For the treated group, combination therapy began on day 0 with external beam radiation to the tumor surface (12 gray) followed by intraperitoneal administration of *α*-CTLA-4 (200 *μ*g) on days 2, 5, and 8 and intratumoral administration of Hu14.18-IL2 (50 *μ*g) a monoclonal anti-GD2 antibody fused to IL2 on days 5 to 9. For the vehicle group, matched PBS injections were administered at the same volumes and frequency as *α*-CTLA-4 and Hu14.18-IL2 in the treated mice, and no radiation was administered. SHG imaging of tumor collagen was performed on pretreatment mice on day 0 and both vehicle-treated and treated mice on days 6, 9, and 12. (b) B78 tumor growth was followed. On the indicated days of imaging, each mouse was anesthetized, and tumor skin flap surgery was performed where dermal and subcutaneous skin layers were gently cut away from the peritoneum revealing the tumor with intact vasculature. The tumor and skin flap were placed on a glass slide for SHG imaging and the mouse on a specially designed microscope stage. Throughout *in vivo* imaging, each mouse was kept under anesthesia and inside a heating chamber that enclosed the microscope stage. All imaging experiments were terminal, with new mice being imaged at each time point. (c) The acquired SHG collagen images were then analyzed using CurveAlign and CT-FIRE software to quantify morphological and phenotypic changes. Qualitative analysis of the collagen images was also performed to evaluate curliness and morphology over time.

Mice were randomized into treatment groups when tumors reached enrollment size (~150 mm^3^), which typically required 3 to 4 weeks of *in vivo* growth. The first day of treatment is defined here as “day 0” [Fig. 1(a)]. On day 0, external beam radiation therapy was delivered to the tumor surface of treated mice only, using an X-RAD 320 cabinet irradiator system (Precision X-Ray, North Branford, Connecticut, United States). Mice were immobilized using custom lead jigs that exposed only the dorsal right flank. Radiation was delivered in one fraction to a maximum dose of 12 Gray (Gy). Systemic mouse *α*-CTLA-4 antibody was administered to treated mice once daily on days 2, 5, and 8 via intraperitoneal injections of 200 *μ*g in 200 *μ*L PBS [Fig. 1(a)]. The *α*-CTLA-4 antibody was provided by Bristol-Meyers Squibb (Redwood City, California, United States). Hu14.18-IL2 IC, a monoclonal anti-GD2 antibody fused to IL2 cytokine, was administered to treated mice once daily on days 5 to 9 via intratumoral injections of 50 *μ*g in 100 *μ*L PBS [Fig. 1(a)]. The Hu14.18-IL2 antibody was provided by AnYxis Immuno-Oncology GmbH (Vienna, Austria). Vehicle-treated mice were injected once daily on days 2, 5, and 8 via intraperitoneal injections of 200 *μ*L PBS and once daily on days 5 to 9 via intratumoral injections of 100 *μ*L PBS. No external beam radiation therapy was administered to vehicle-treated mice. Pretreatment mice received no PBS or immunotherapy injections and no external beam radiation therapy.

#### 2.1.3 Intravital tumor imaging

Intravital imaging of the mouse melanoma tumors was performed in pretreatment mice (*n* = 4), vehicle-treated mice (*n* = 6), and treated mice (*n* = 6). Imaged mice were 50% male and 50% female. Images were acquired on days 0, 6, 9, and 12 of therapy [Fig. 1(a)], in two independent experiments, to capture temporal collagen changes throughout the course of treatment. Day 0 images, the pretreatment group, are used as a baseline comparison for both vehicle and immunotherapy-treated groups. Immediately prior to tumor imaging, skin flap surgery exposed flank tumors. Mice were anesthetized with isoflurane, and then, the skin around the tumor was cut into a flap and separated from the body cavity so that the tumor laid flat on the imaging stage while still connected to the vasculature.^60-62^ Mice were placed on a specialized microscope stage for imaging and kept in a heating chamber (air maintained at 37°C) during imaging. An imaging dish insert and PBS for coupling were used with surgical tape to secure skin flap tumors [Fig. 1(b)]. All imaging experiments were terminal, with new mice being imaged at each time point. The skin flap method allowed easy tumor access for intratumoral immunotherapy administration on days 5 to 9 and eliminated any immune response related to the flank window method itself.

### 2.2 Collagen SHG Imaging

Collagen SHG images were captured with a custom-built multi-photon microscope (Bruker, Billerica, Massachusetts, United States) using an ultrafast femtosecond laser (InSight DSC, Spectra Physics, Santa Clara, California, United States) with linear polarization. We acknowledge that without a circular polarization compensator, all collagen fibers may not be excited equally here. Images of collagen fibers were detected using a bandpass filter of 514/30 nm with a 1041 nm excitation (typical power 2.1 to 3.4 mW). All images were acquired with a 40 × /1.15 NA water-immersion objective with a 0.59 to 0.6 mm working distance (Nikon) at 512 × 512 pixel resolution, 4.8 *μ*s pixel dwell time, 32 average frames, and an optical zoom of 1.0. The pixel dimension of the microscope is 586 nm, and the resolution limit is 227 nm, indicating we are sampling 2.5-fold above the resolution limit. SHG images were acquired to sample collagen changes during therapy across 2 to 7 fields of view and multiple depths (in the range of 10 to 127 *μ*m in the *z* dimension) within each tumor.

### 2.3 Collagen SHG Image Analysis

Several quantitative parameters were extracted using two packages of curvelet-based analysis software: CurveAlign and CT-FIRE [Fig. 1(c)]. CurveAlign can perform bulk analysis of collagen at the FOV level, where output metrics depend on all curvelets within the FOV (coefficient of alignment and density) and describe the FOV as a whole. CT-FIRE can quantify collagen metrics independent of other curvelets at an individual collagen fiber level (straightness, length, width). In addition, CT-FIRE individual collagen fiber metrics can be averaged to investigate FOV level differences. We chose to investigate both bulk FOV and single-fiber collagen changes within this dataset to provide a comprehensive view of collagen morphology changes. We anticipate that this comprehensive analysis will improve the overall accuracy of the data as tumor heterogeneity is expected.

#### 2.3.1 CurveAlign analysis

CurveAlign^6,20,29-31^ was used to quantify bulk collagen features, including the collagen coefficient of alignment and density from the SHG images. These metrics can only be calculated at a FOV level as they are dependent on all curvelets within the FOV. Eight-bit SHG collagen images were imported into CurveAlign, and the fraction of coefficients to keep was set to 0.04. CurveAlign was used to calculate the coefficient of alignment for each FOV on a scale between 0 and 1, where 0 is unaligned and 1 is fully aligned collagen fibers. Along with the coefficient of alignment, CurveAlign was also used to calculate the density of collagen within the FOV. Density calculations required a region of interest (ROI) analysis. For our purposes, we set the entire FOV to be the ROI. The threshold was set to 50 based on optimization calculations to remove as much background as possible, and the CurveAlign density parameter was calculated as the number of pixels within the FOV that were above the threshold. To calculate the true density, the CurveAlign density was divided by the total area of the FOV in pixels, resulting in a true density value between 0 and 1 where 0 is no pixels within the FOV containing collagen fibers, and 1 is every pixel within the FOV contains collagen fibers. CurveAlign was used to analyze images at a FOV level for pretreatment mice (*n* = 4 mice, 18 FOV), vehicletreated mice (*n* = 6 mice, 32 FOV), and immunotherapy-treated mice (*n* = 6 mice, 29 FOV).

#### 2.3.2 CT-FIRE analysis

The CT-FIRE^20,29,30^ module was used to extract single-fiber features such as collagen straightness, length, and width at an individual collagen fiber level. CT-FIRE was designed to operate at a single curvelet level. Collagen straightness was defined here as the distance between the collagen fiber endpoints divided by the distance along the path of the fiber. A straightness value of 1 indicates a perfectly straight fiber, while a straightness value of 0 indicates a highly curly fiber. The length parameter measured the distance along the fiber from one end to the other while the width was the average width along the fiber. Both length and width were measured in pixels. CT-FIRE was used to analyze pretreatment mice single collagen fibers (*n* = 4 mice, 4282 single fibers), vehicletreated mice single collagen fibers, (*n* = 6 mice, 7081 single fibers), and immunotherapy-treated mice single collagen fibers (*n* = 6 mice, 6749 single fibers) at the individual curvelet level. In addition, the single curvelet features were averaged to examine FOV level differences for comparison.

#### 2.3.3 Qualitative curliness scoring

**Fig. 2:**
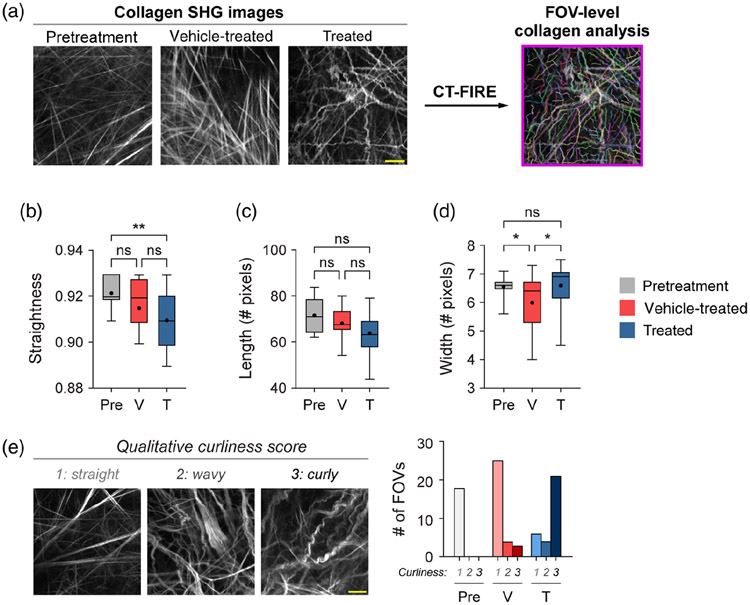
FOV-level analysis of collagen features by treatment group with CT-FIRE. (a) Representative *in vivo* SHG collagen images in B78 mouse melanoma tumors either from pretreatment, vehicle-treated, or treated mice. FOV-level collagen analysis was performed using CT-FIRE. (b) FOV-level collagen straightness from pretreatment, vehicle-treated, and treated mice (mean straightness: 0.92 pretreatment, 0.92 vehicle-treated, 0.91 treated). A straightness value of 1 indicates a perfectly straight fiber, while a straightness value of 0 indicates a highly curly fiber. (c) FOV-level collagen length from pretreatment, vehicle-treated, and treated mice (mean length: 72 pretreatment, 68 vehicle-treated, 64 treated). (d) FOV-level collagen width from pretreatment, vehicle-treated, and treated mice (mean width: 6.6 pretreatment , 6.0 vehicle-treated, 6.6 treated). Box and whisker: median ± min / max, mean = dot. (e) Qualitative curliness scoring of SHG images from pretreatment, vehicle-treated, and treated mice (*n* = 79 FOV) into three bins: 1 straight, 2 wavy, and 3 curly. Example images with their corresponding qualitative curliness score are shown. *n* = 4 to 6 mice per treatment group, pretreatment images *n* = 18 FOV, vehicle images *n* = 32 FOV, treated images *n* = 29 FOV. ANOVA with Tukey’s HSD, *p < 0.05, **p < 0.01. FOV, field of view. Scale bar 50 *μ*m.

SHG images were qualitatively scored, similar to the standard clinical practice of scoring tumor histology slides, to determine macroscopic collagen changes. This fast, low-barrier analysis provided supportive data in a clinically relevant format, to complement the quantitative collagen metrics measured with CurveAlign and CT-FIRE. Blinded qualitative curliness scoring of SHG images was performed by scoring images into bins of 1, 2, or 3 based on whether the majority of the macroscopic collagen fibers within each FOV were very straight and aligned (score 1), wavy and heterogeneous (score 2), or very curly and tortuous (score 3). Representative images for each score are shown in Fig. 2(e).

### 2.4 Immunofluorescence

Excised mouse melanoma tumor tissues were formalin-fixed and paraffin-embedded for antibody staining with a fluorescent marker of fibroblasts [*α*-smooth muscle actin (α-SMA), abcam SP171 ab150301]. Embedded sections were deparaffinized and hydrated prior to antigen retrieval and placement in a blocking solution. Next, the primary antibody was applied upon removal of the blocking solution at the following dilution and incubation time: *α*-SMA—1:200 for 15 min at room temperature. A secondary rabbit antibody was then added following the primary antibody incubation at 1:500 for 10 min at room temperature. Then, a staining dye was added after secondary antibody washes: *α*-SMA—Opal-dye 520 (Akoya Biosciences OP-001001, Marlborough, Massachusetts, United States) at 1:100. Finally, stained sections were incubated in DAPI for 5 min at room temperature for nuclear labeling and mounted on coverslips for imaging. Imaging was performed at 20× using a Vectra multispectral imaging system (Akoya Biosciences), and a spectral library was generated to separate spectral curves for each fluorophore. The resulting images were analyzed using Nuance and inForm software (Akoya Biosciences).

### 2.5 Statistical Analysis

**Fig. 3:**
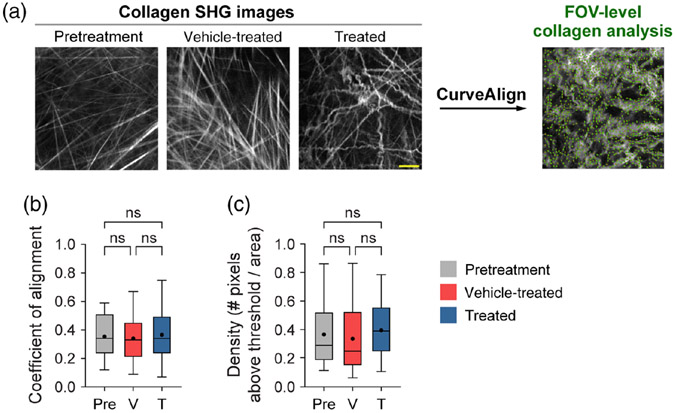
FOV-level analysis of collagen coefficient of alignment and density changes by treatment group using CurveAlign. (a) Representative *in vivo* SHG collagen images in B78 mouse melanoma tumors either from pretreatment, vehicle-treated, or treated mice. FOV-level collagen analysis was performed using CurveAlign. (b) FOV-level collagen coefficient of alignment from pretreatment, vehicle-treated, and treated mice (mean coefficient of alignment: 0.35 pretreatment, 0.34 vehicle-treated, 0.37 treated). For the coefficient of alignment, 0 indicates fibers are unaligned, and 1 indicates fibers are fully aligned. (c) FOV-level collagen density from pretreatment, vehicle-treated, and treated mice (mean density: 0.37 pretreatment, 0.34 vehicle-treated, 0.40 treated). Box and whisker: median ± min / max, mean = dot. *n* = 4 to 6 mice per treatment group, pretreatment images *n* = 18 FOV, vehicle images *n* = 32 FOV, treated images *n* = 29 FOV. ANOVA with Tukey’s HSD. FOV, field of view. The scale bar is 50 *μ*m.

**Fig. 4:**
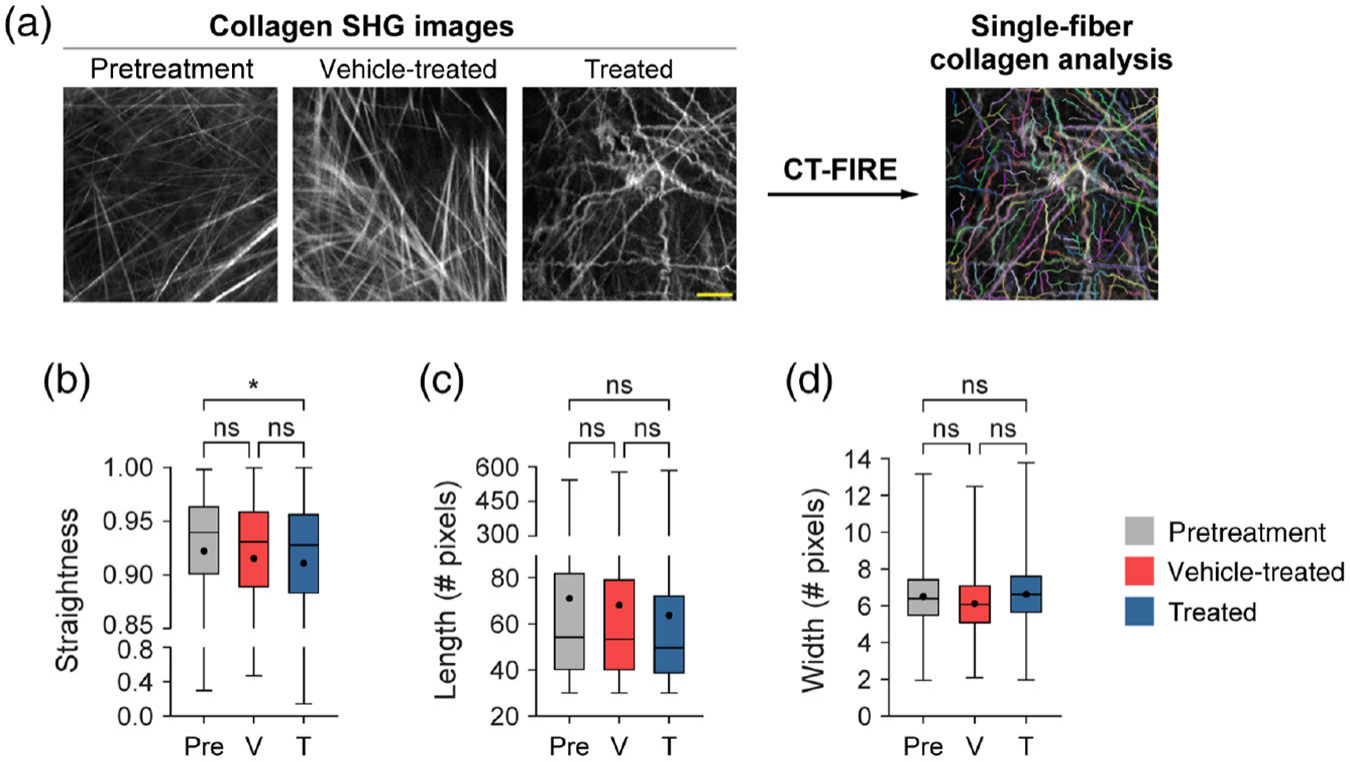
Single-fiber level analysis of collagen features by the treatment group with CT-FIRE. (a) Representative *in vivo* SHG collagen images in B78 mouse melanoma tumors either from pretreatment, vehicle-treated, or treated mice. Single-fiber collagen analysis was performed using CT-FIRE. (b) Single-fiber level collagen straightness from pretreatment, vehicle, and treated mice (mean straightness: 0.92 pretreatment, 0.92 vehicle-treated, 0.91 treated). A straightness value of 1 indicates a perfectly straight fiber, while a straightness value of 0 indicates a highly curly fiber. (c) Single-fiber level collagen length from pretreatment, vehicle-treated, and treated mice (mean length: 71 pretreatment, 68 vehicle-treated, 64 treated). (d) Single-fiber level collagen width from pretreatment, vehicle-treated, and treated mice (mean width: 6.5 pretreatment, 6.2 vehicle-treated, 6.7 treated). Box and whisker: median ± min / max, mean = dot. *n* = 4 to 6 mice per treatment group, pretreatment curvelets *n* = 4282, vehicle curvelets *n* = 7081, treated curvelets *n* = 6749. ANOVA with Tukey’s HSD, *p < 0.05. The scale bar is 50 *μ*m.

**Fig. 5:**
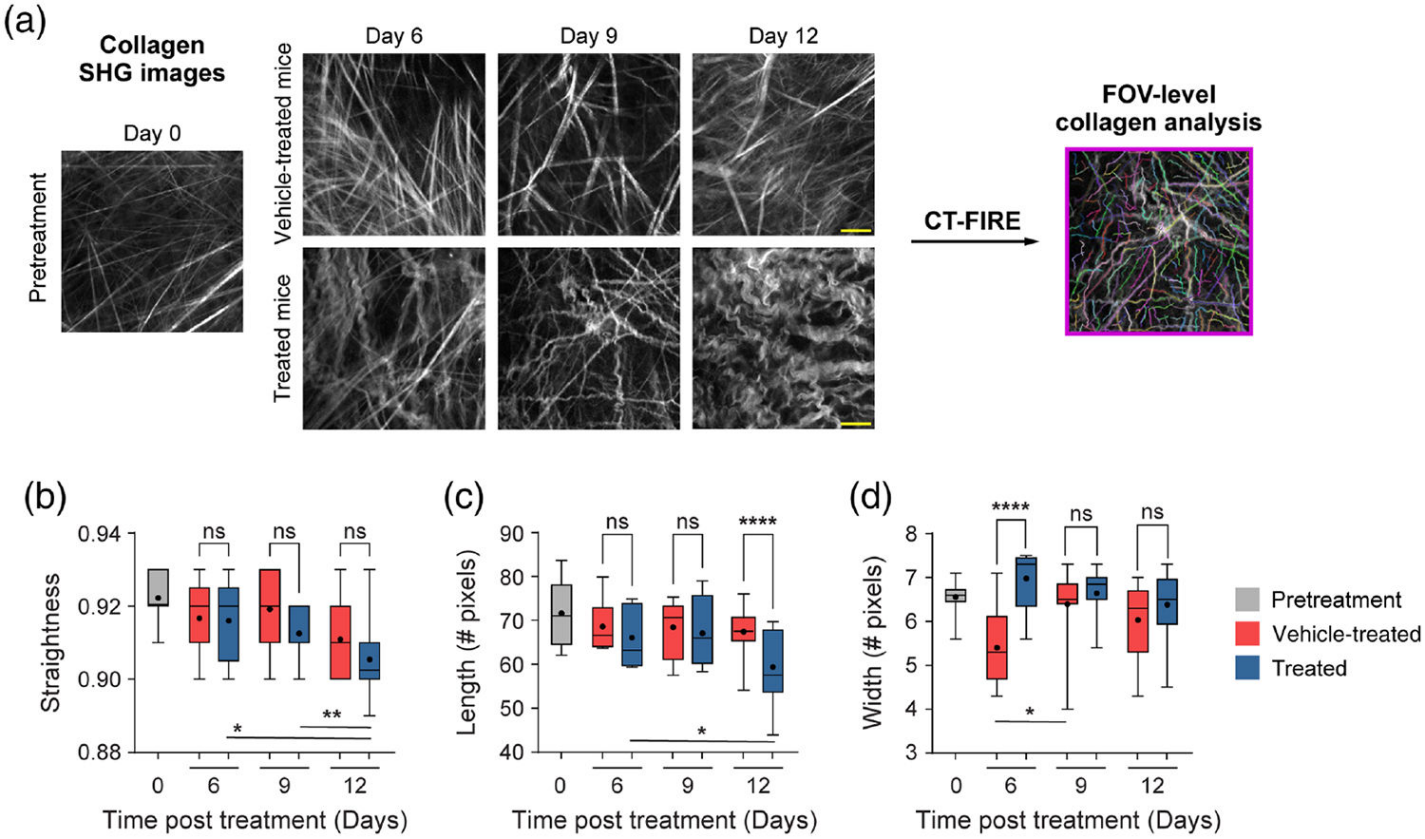
FOV-level analysis of collagen features over time with treatment using CT-FIRE. (a) Representative *in vivo* SHG collagen images in B78 mouse melanoma tumors from day 0 pretreatment mice and days 6, 9, and 12 vehicletreated and treated mice. FOV level collagen analysis was performed using CT-FIRE. (b) FOV-level collagen straightness from pretreatment, vehicletreated, and treated mice over time with treatment (mean straightness vehicle: treated day 6 0.92:0.92, day 9 0.92:0.91, day 12 0.91:0.91). A straightness value of 1 indicates a perfectly straight fiber, while a straightness value of 0 indicates a highly curly fiber. (c) FOV-level collagen length from pretreatment, vehicle-treated, and treated mice over time with treatment (mean length vehicle: treated day 6 69:66, day 9 68:67, day 12 67:59, mean length treated: treated days 9 to 12 67:59). (d) FOV-level collagen width from pretreatment, vehicle-treated, and treated mice over time with treatment (mean width vehicle: treated day 6 5.4:7.0, day 9 6.4:6.6, day 12 6.0:6.4, mean width vehicle: vehicle days 6 to 9 5.4:6.4). Note, the day 9 vehicle median value is present but very close to the minimum value. Box and whisker: median ± min / max, mean = dot. *n* = 4 to 6 mice per treatment group, pretreatment images *n* = 18 FOV, vehicle images *n* = 32 FOV, treated images *n* = 29 FOV. ANOVA with Tukey’s HSD, *p < 0.05, **p < 0.01, ****p < 0.0001. FOV, field of view. The scale bar is 50 *μ*m.

**Fig. 6:**
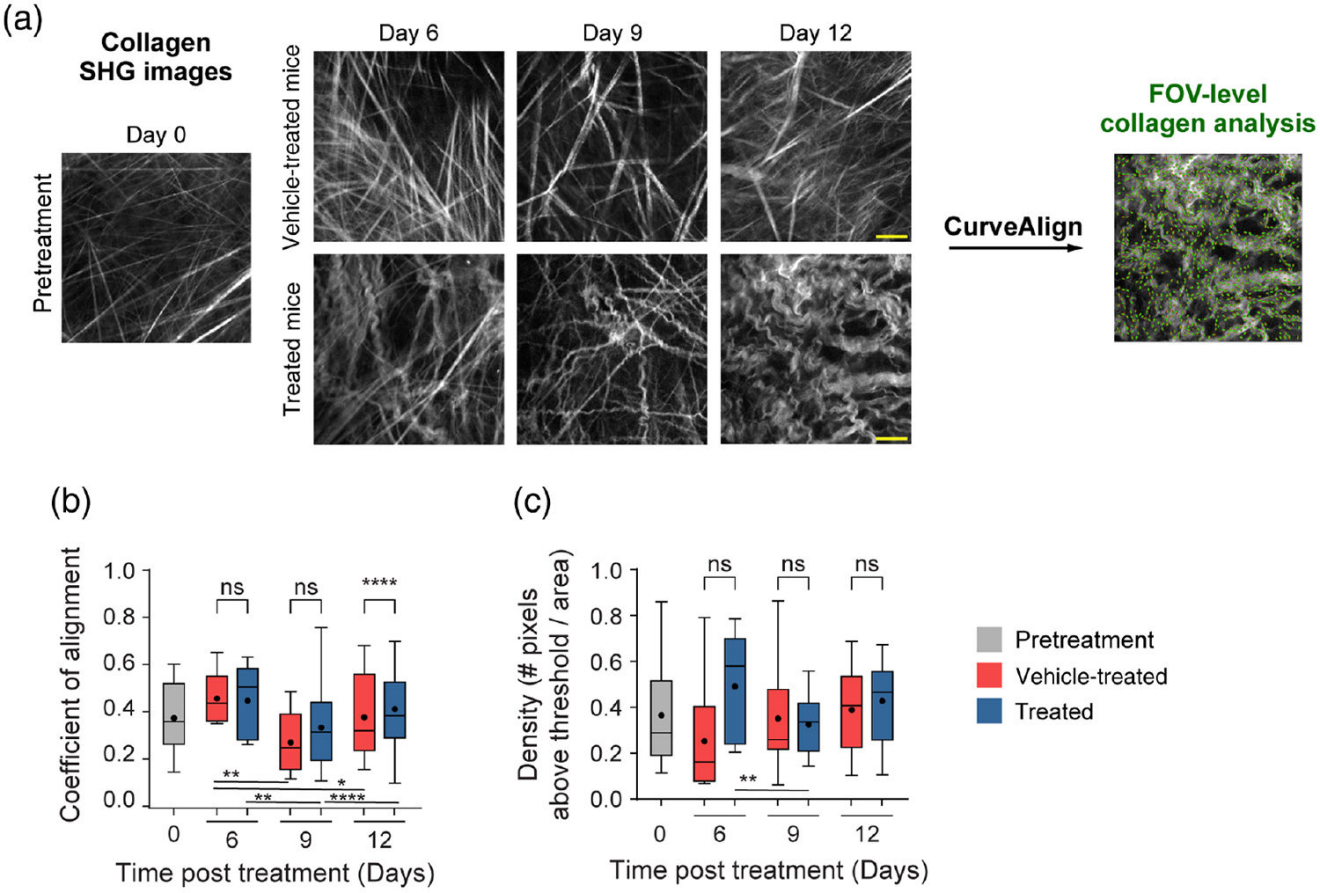
FOV-level analysis of collagen coefficient of alignment and density changes over time with treatment using CurveAlign. (a) Representative *in vivo* SHG collagen images in B78 mouse melanoma tumors from day 0 pretreatment mice and days 6, 9, and 12 vehicle-treated and treated mice. FOV-level collagen analysis was performed using CurveAlign. (b) FOV-level collagen coefficient of alignment from pretreatment, vehicle-treated, and treated mice over time with treatment (mean coefficient of alignment vehicle: treated day 6 0.44:0.43, day 9 0.25:0.31, day 12 0.36:0.39, mean coefficient of alignment vehicle: vehicle days 6 to 9 0.44:0.25). For the coefficient of alignment, 0 indicates fibers are unaligned, and 1 indicates fibers are fully aligned. (c) FOV-level collagen density from pretreatment, vehicle-treated, and treated mice over time with treatment (mean density vehicle: treated day 6 0.25:0.49, day 9 0.35:0.33, day 12 0.39:0.43). Box and whisker: median *±* min / max, mean = dot. *n* = 4 to 6 mice per treatment group, pretreatment images *n* = 18 FOV, vehicle images *n* = 32 FOV, treated images *n* = 29 FOV. ANOVA with Tukey’s HSD, *p < 0.05, **p < 0.01, ****p < 0.0001. FOV, field of view. The scale bar is 50 *μ*m.

**Fig. 7:**
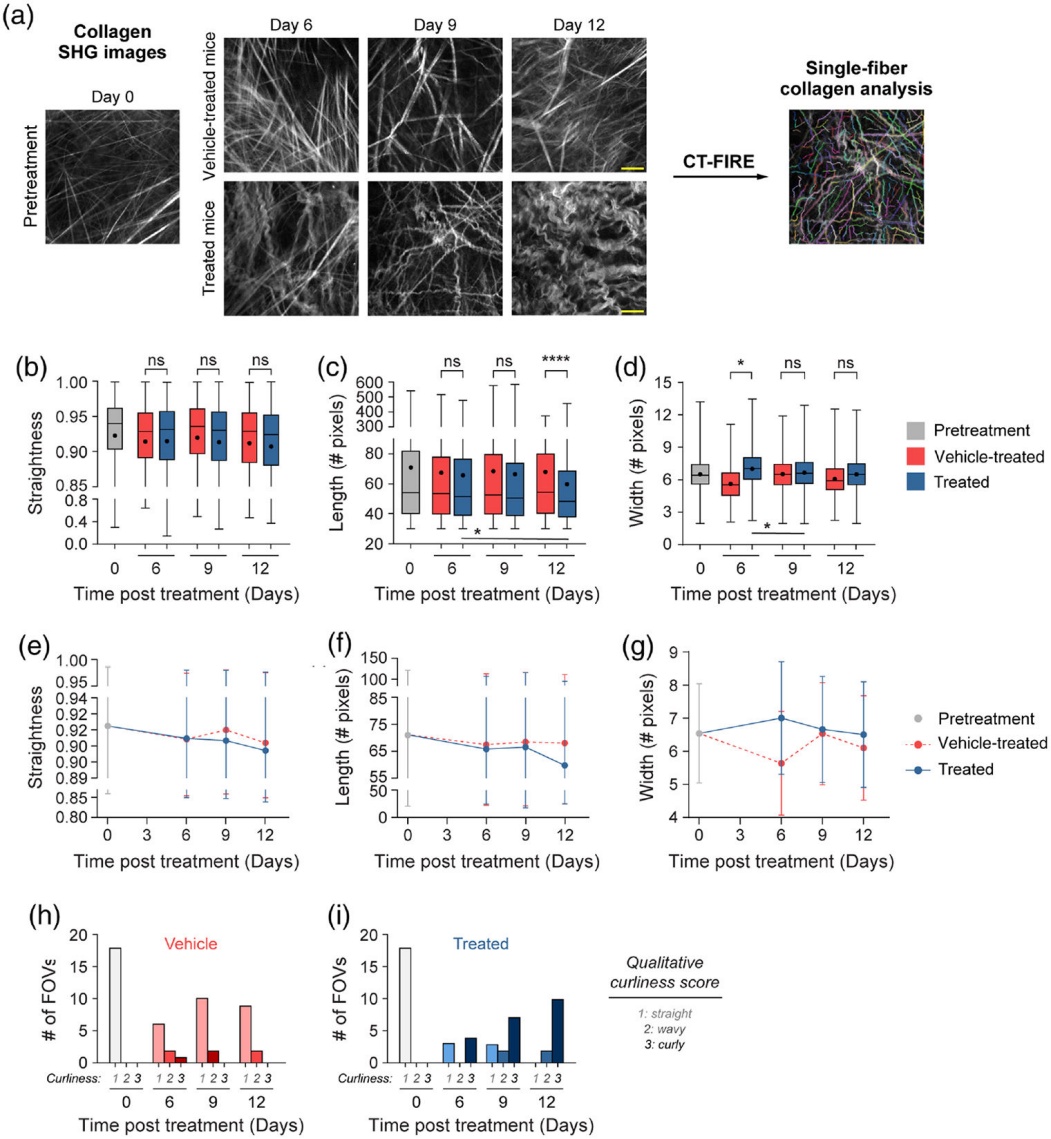
Single-fiber level analysis of collagen features over time with treatment using CT-FIRE. (a) Representative *in vivo* SHG collagen images in B78 mouse melanoma tumors from day 0 pretreatment mice and days 6, 9, and 12 vehicletreated and treated mice. Single-fiber analysis was performed using CT-FIRE. (b) Single-fiber collagen straightness from pretreatment, vehicletreated, and treated mice over time with treatment (mean straightness vehicle: treated day 6 0.91:0.91, day 9 0.92:0.91, day 12 0.91:0.91, mean straightness vehicle: vehicle days 9 to 12 0.92:0.91, mean straightness treated: treated days 6 to 12 0.91:0.91, days 9 to 12 0.91:0.91). A straightness value of 1 indicates a perfectly straight fiber, while a straightness value of 0 indicates a highly curly fiber. (c) Single-fiber collagen length from pretreatment, vehicle-treated, and treated mice over time with treatment (mean length vehicle: treated day 6 67:66, day 9 68:67, day 12 68:60, mean length treated: treated days 6 to 12 66:67, days 9 to 12 67:60). (d) Single-fiber collagen width from pretreatment, vehicle-treated, and treated mice over time with treatment (mean width vehicle: treated day 6 5.6:7.0, day 9 6.5:6.7, day 12 6.1:6.5, mean width vehicle: vehicle days 6 to 9 5.6:6.5, days 9 to 12 6.5:6.1, days 6 to 12 5.6:6.1, mean width treated: treated days 6 to 9 7.0:6.7, days 9 to 12 6.7:6.5, days 6 to 12 7.0:6.5). Box and whisker: median ± min / max, mean = dot. (e)–(g) Curves for single-fiber collagen straightness, length, and width over time with treatment. Curves: mean ± SD. (h)–(i) Qualitative curliness scoring of SHG images from pretreatment, vehicle-treated, and treated mice over time with treatment (*n* = 79 FOV) into three bins: 1 straight, 2 wavy, and 3 curly. *n* = 4 to 6 mice per treatment group, pretreatment curvelets *n* = 4282, vehicle curvelets *n* = 7081, treated curvelets n = 6749. ANOVA with Tukey’s HSD, *p < 0.05, ****p < 0.0001. The scale bar is 50 *μ*m.

Analysis of variance (ANOVA) was conducted to evaluate treatment group differences in collagen parameters between every combination of the three treatment groups (pretreatment, vehicletreated, and treated), differences over time between vehicle-treated and treated groups on each imaging day, and differences in fibroblast numbers over time. Group and day were included as the main factors in these analyses. To conduct comparisons between days within groups, sliced two-way interaction contrasts were constructed. A compound symmetry correlation structure was used to account for correlations between subsamples within each animal. Tukey’s Honestly Significant Difference (HSD) method was used to control the type I error when conducting multiple pairwise comparisons. Model assumptions were validated by examining residual plots [Figs. 2-7 and Fig. S1 in the Supplementary Material, SAS Software, SAS Institute, Cary, North Carolina, United States, version 9.4]. Collagen results are represented as box and whisker plots showing median ± min / max, with the mean represented as a dot. Collagen curves are represented as mean ± standard deviation. Fibroblast results are represented as scatter plots showing mean ± standard deviation. All reported *p*-values are two-sided, and *p* < 0.05 was used to define statistical significance.

## 3 Results

### 3.1 FOV-Level Collagen Analysis Separates Mice by Treatment Group

Mouse melanoma tumors were imaged *in vivo* in pretreatment mice, vehicle-treated mice with no radiation (referred to as vehicle-treated mice), and radiation plus immunotherapy-treated mice (referred to as treated mice). CT-FIRE was used to calculate curvelet-based collagen changes at the FOV level from SHG images of all three treatment groups [Fig. 2(a)]. For the quantified data in Figs. 2-3, *n* represents the number of FOV. Representative *in vivo* SHG images show striking collagen fiber morphology differences across treatment groups [Fig. 2(a)]. Quantitatively, collagen from treated mice was significantly less straight than collagen from pretreatment mice [Fig. 2(b), **p < 0.01]. Straightness differences between treated and vehicle-treated mice were not statistically significant although treated mice appeared to have less straight collagen [Fig. 2(b)]. No significant difference in straightness was seen between vehicle and pretreatment mice either though vehicle-treated mice trended toward less straight collagen [Fig. 2(b), *p* = 0.16]. Second, no significant differences in collagen length were found although a trend was observed where treated mice had shorter collagen fibers compared with pretreatment mice [Fig. 2(c), *p* = 0.16]. Third, collagen from treated mice was significantly wider than vehicletreated mice [Fig. 2(d), *p < 0.05] though not significantly different compared with pretreatment mice. Collagen from vehicle-treated mice was also thinner compared with pretreatment mice [Fig. 2(d), *p < 0.05]. Finally, qualitative curliness scoring of collagen images at the FOV level was performed by scoring of blinded images into bins of 1, 2, or 3 based on whether the collagen fibers were very straight (score 1), wavy (score 2), or very curly (score 3). Representative images for each score are shown in Fig. 2(e). Qualitative curliness scoring illustrated that pretreatment mice images were all scored at 1, indicating homogenous, very straight collagen across all FOV [Fig. 2(e)]. By contrast, vehicle-treated mice showed some heterogeneity with these images scored mostly as 1 (*n* = 25 FOV, 78%) with a few images scored as 2 (*n* = 4 FOV) or 3 (*n* = 3 FOV) [Fig. 2(e)]. Treated mice also showed heterogeneity with very few images scored as 1 (*n* = 6 FOV) or 2 (*n* = 4 FOV) and most images scored as 3 (*n* = 21 FOV, 68%), indicating very curly collagen [Fig. 2(e)]. Overall, qualitative curliness scoring resulted in mostly straight collagen in pretreatment (100%) and vehicle-treated mice (78%) with mostly curly collagen in treated mice (68%) [Fig. 2(e)]. Conversely, FOV level changes in collagen calculated with CurveAlign [Fig. 3(a)] showed no significant difference in the collagen coefficient of alignment between treatment groups [Fig. 3(b)]. Similarly, CurveAlign found no significant differences in collagen density across the three treatment groups at a FOV level, although treated mice appeared to have the highest density [Fig. 3(c)].

### 3.2 CT-FIRE Assesses Changes in Single-Fiber Collagen Morphology and Separates Mice by Treatment Group

Quantitative curvelet-based collagen analysis was performed with CT-FIRE to assess changes at the single-fiber level [Fig. 4(a)], in contrast to the FOV-level analysis described in Sec. 3.1. For the quantified data in Fig. 4, *n* represents the number of single fibers. Single collagen fibers from treated mice were significantly less straight than collagen from pretreatment mice only [Fig. 4(b), *p < 0.05]. Single collagen fibers from vehicle-treated mice trended toward being less straight than collagen from pretreatment mice though this difference was not significant [Fig. 4(b), *p* = 0.13]. No significant differences in single collagen fiber length were observed across all treatment groups [Fig. 4(c)]. Similarly, no significant differences in single collagen fiber width were observed across all treatment groups although single collagen fibers from treated mice trended toward being wider compared with vehicle single collagen fibers [Fig. 4(d), *p* = 0.07]. A trend was also observed where single collagen fibers from vehicle-treated mice trended toward being thinner compared with pretreatment single collagen fibers [Fig. 4(d), *p* = 0.19]. Overall, treated mice had collagen fibers that were curlier, shorter in length, and wider compared with vehicle-treated and pretreatment mice.

### 3.3 CT-FIRE and CurveAlign Show Time-Dependent Collagen Morphology Changes at the FOV Level

FOV-level analysis for each day of treatment was performed using CT-FIRE and CurveAlign to extract time and treatment-dependent changes. Representative *in vivo* SHG images show striking collagen fiber morphology differences across treatment groups and time-course [Fig. 5(a)]. For the quantified data in Figs. 5-6, *n* represents the number of FOV. With CT-FIRE analysis, vehicle mouse melanoma tumors, PBS injections only with no radiation, showed aligned, straight collagen fibers at days 6, 9, and 12 [Fig. 5(a)] that mirror the day 0 pretreatment phenotype. By contrast, treated mouse melanoma tumors, radiation and immunotherapy treated, showed curly, tortuous collagen fibers at days 6, 9, and especially 12 [Fig. 5(a)]. Surprisingly, no significant changes in collagen straightness were seen across the treatment course, at the FOV level, when comparing treated and vehicle-treated mice [Fig. 5(b)]. Treated mice appeared to have less straight, curlier collagen compared with vehicle-treated mice on days 9 and 12, although these differences were insignificant. Treated mice showed significantly shorter collagen fiber length compared with vehicle-treated mice at day 12 only [Fig. 5(c), ****p < 0.0001]. Finally, treated mice exhibited significantly wider collagen fibers compared with vehicle-treated mice at day 6 only [Fig. 5(d), ****p < 0.0001]. Collagen changes within a treatment group over time were also observed at the FOV level. Within the treated group, collagen fibers became significantly curlier with time [Fig. 5(b), days 6 to 12 *p < 0.05, days 9 to 12 **p < 0.01] and their length shortened significantly with time [Fig. 5(c), days 6 to 12 *p < 0.05, trend observed days 9 to 12 *p* = 0.07] with a trend toward thinner fibers with time [Fig. 5(d), days 6 to 9 *p* = 0.17]. Within the vehicle group, collagen fiber width increased significantly with time [Fig. 5(d), days 6 to 9 *p < 0.05] with no differences in straightness or length [Figs. 5(b), 5(c)]. With CurveAlign FOV-level analysis (Fig. 6), the collagen coefficient of alignment was significantly different on day 12 only when comparing treated and vehicle-treated mice [Fig. 6(b), ****p < 0.0001]. Unexpectedly, the day 12 coefficient of alignment measurements indicate that treated mouse fibers are more aligned than vehicle-treated ones [Fig. 6(b)]. No significant differences in collagen density between treated and control mice over time were observed though treated mice did trend toward higher density on day 6 [Fig. 6(c), vehicle day 6 versus treated day 6 *p* = 0.06]. Within the vehicle group, the collagen coefficient of alignment decreased significantly over time from days 6 to 9 and days 6 to 12 with a trending increase from days 9 to 12 [Fig. 6(b), days 6 to 9 **p < 0.01, days 6 to 12 *p < 0.05, days 9 to 12 *p* = 0.10] and no changes in density [Fig. 6(c)]. Within the treated group, the collagen coefficient of alignment changed significantly over time with a decrease on days 6 to 9 and an increase on days 9 to 12 [Fig. 6(b), days 6 to 9 **p < 0.01, days 9 to 12 ****p < 0.0001]. Collagen density also changed within the treated group over time with a significant decrease observed from days 6 to 9 and a trending increase from days 9 to 12 [Fig. 6(c), days 6 to 9 **p < 0.01, days 9 to 12 *p* = 0.17].

### 3.4 CT-FIRE Shows Time-Dependent Changes in Single-Fiber Collagen Morphology

Finally, single-fiber level analysis was performed for each day of treatment using CT-FIRE to extract time and treatment-dependent changes [Fig. 7(a)]. For the quantified data in Figs. 7(b)-7(d), *n* represents the number of single fibers; Figs. 7(h)-7(i) is at the FOV level. No significant differences in collagen single-fiber straightness were observed with time when comparing treated and vehicle-treated mice [Fig. 7(b)]. Treated mice exhibited significantly shorter collagen fibers on day 12 of treatment compared with vehicle-treated mice [Fig. 7(c), ****p < 0.0001]. In addition, collagen fibers from treated mice were significantly wider compared with vehicle-treated mice on day 6 of treatment [Fig. 7(d), *p < 0.05]. Single-fiber collagen changes were also observed within treatment groups over time. Within the treated group, a trend toward curlier fibers was observed between days 9 and 12 [Fig. 7(b), *p* = 0.065]. Treated collagen fiber length decreased significantly between days 6 and 12 with a continued downward trend between days 9 and 12 [Fig. 7(c), days 6 to 12 *p < 0.05, days 9 to 12 *p* = 0.07]. Treated single-fiber width also decreased significantly between days 6 and 9 with a downward trend observed between days 6 and 12 [Fig. 7(d), days 6 to 9 *p < 0.05, days 6 to 12 *p* = 0.17]. Within the vehicle group, no significant changes were found in straightness, length, or width over time [Figs. 7(b)-7(d)]. A summary of collagen changes over time across treatment groups highlights that treated mouse collagen straightness and length decreased the most on day 12 [Figs. 7(e)-7(f)], while width increased the most on day 6 [Fig. 7(g)]. Qualitative scoring of collagen images shows that pretreatment mouse tumors were scored exclusively as 1, indicating a straight phenotype [Figs. 7(h)-7(i)]. Similarly, 78% of vehicle-treated mouse tumor images were scored as 1, a straight phenotype, across all treatment days [Fig. 7(h)]. As a result, very few vehicle-treated mouse tumor images were scored as 2 (*n* = 4 FOV) or 3 (*n* = 3 FOV) across treatment time [Fig. 7(h)]. By contrast, treated mouse tumors were qualitatively scored mostly as 3 (68%), a curly phenotype, across all treatment days [Fig. 7(i)]. Only a few images (*n* = 4 FOV) were scored as 2, wavy, or (*n* = 6 FOV) 1, straight [Fig. 7(i)]. Interestingly, the few treated mouse images that were scored as 1, straight, were only from days 6 and 9 post-treatment, with all collagen fibers scored as wavy or curly (2 or 3) by day 12 [Fig. 7(i)].

### 3.5 Immunofluorescence Shows *α*-SMA + Tumor Fibroblast Changes with Treatment

Following imaging studies, paired mouse melanoma tumor tissues were excised, formalin-fixed, and paraffin-embedded for antibody staining with a fluorescent marker of fibroblasts: *α*-SMA. Representative immunofluorescence images showed fibroblast populations within melanoma tumors fluctuated during treatment [Fig. S1(a) in the Supplementary Material]. Interestingly, *α*-SMA+ fibroblast numbers significantly increased early in treatment on day 6 [Fig. S1(b) in the Supplementary Material, ****p < 0.0001] with a trend toward a decrease in fibroblast numbers on day 9 [Fig. S1(b) in the Supplementary Material, *p* = 0.051]. For the quantified data in Fig. S1 in the Supplementary Material, *n* represents the number of fibroblasts per FOV.

## 4 Discussion

During tumorigenesis, cancer cells and CAFs secrete excess MMPs and collagen, which often leads to the reconstruction, linearization, and remodeling of collagen in the tumor microenvironment.^3^ This remodeling may be especially relevant for melanoma patients whose skin is 70% collagen and whose tumors are often fibroblast-rich.^34-37,63^ Though SHG imaging has enabled insights into collagen trends in breast and pancreatic cancer, few studies have performed SHG of collagen in melanoma.^22,54,55^ To our knowledge, this is the first study to quantify *in vivo* mouse melanoma tumor collagen reorganization during immunotherapy treatment using label-free SHG imaging. We quantified six collagen morphology features during combination radiotherapy and immunotherapy or PBS vehicle using CurveAlign and CT-FIRE software. We examined collagen reorganization and phenotypic changes with temporal context at both FOV and single-fiber levels. We found that collagen length and width changes were the most sensitive parameters to distinguish treated and control tumors. Temporally, collagen length and curliness changes occurred primarily on days 9 and 12 of treatment, while collagen width changes occurred primarily on day 6 of treatment.

*In vivo* SHG imaging provided a clear visualization of collagen fibers within mouse tumors. We observed distinct collagen fiber morphology that varied by treatment group and time. Our imaging captured collagen reorganization that occurred only in the radiation and immunotherapy-treated mice during treatment. Overall, we showed that live SHG imaging of mouse melanoma tumors produced high-contrast images where mice could be categorized by the treatment group based on collagen phenotype that changed during therapy.

We reported FOV-level collagen changes using features from both CurveAlign and CT-FIRE. Our FOV-level collagen coefficient of alignment measurements performed in CurveAlign was not statistically different across all three groups. When treated and vehicle-treated mice were compared across time, however, treated mice exhibited a surprisingly higher coefficient of alignment on day 12 only which was out of trend with our CT-FIRE data. As the coefficient of alignment is dependent on both the straightness of collagen as well as the fiber orientations in relation to each other, this calculation is most accurate when a defined tumor border can be marked within the FOV.^29^ Our *in vivo* images were acquired within the tumor so no such border was present, which may partly explain the limited effectiveness of this parameter. If desired, we could acquire *in vivo* mouse SHG images at the tumor edge to draw a border within each FOV, which should improve the relevance and accuracy of the coefficient of alignment. Interestingly, our FOV-level density measurements showed that treated mouse tumors appeared to have higher collagen density, but only a trend toward significance was observed on day 6. Though increased collagen density leads to negative outcomes in many cancers, similar findings have been shown in human melanoma and human/rat breast cancer where increased collagen density was associated with better tumor outcomes.^6,55,64^ This increased collagen density in the skin may help prevent tumor cell metastasis, linking it to a healthy phenotype.^2^ By contrast, CTFIRE showed statistically significant differences in straightness and width of collagen fibers from treated mice versus PBS control. At the FOV level, collagen fibers from treated mice versus control mice are curlier or less straight and shorter in length. This indicates that collagen fibers from treated mice are less linearized and may be transitioning toward a healthier phenotype. This shorter collagen characteristic may indicate cleavage of collagen fibers by MMPs from local fibroblasts.^22^ In addition, collagen fibers from treated mice versus PBS control mice are wider, which further supports our assessment that treated mouse tumors contain collagen that is less linearized. Perhaps this increase in width is a result of collagen fibers that are less stretched and less taut. Others have similarly shown that shorter, wider, curlier collagen is a characteristic of healthy human skin compared with basal cell carcinoma biopsies.^65,66^

We also showed single-fiber collagen changes using CT-FIRE. Radiation- and immunotherapy-treated mouse tumors possessed collagen fibers that were significantly less straight and trending toward being shorter and wider compared with PBS control tumors. This treated tumor collagen phenotype is consistent with what is expected for healthy tissue collagen versus TACSs where straightness and alignment are increased.^5,20^ The decrease in straightness or alignment in our treated mouse tumors was also consistent with *in vivo* collagen SHG of mouse healthy ears (less aligned) compared with mouse ears bearing B16-F10 melanoma (more aligned).^54^ In addition, shorter and wider collagen fibers restrict tumor cell invasion, via decreased *β*1 integrins and MMPs associated genes as well as increased E-cadherin on tumor cells, in human and rat breast cancers.^64^ The difference in collagen phenotype between our PBS vehicle tumors and treated mouse tumors may be due to the treatment itself or changes occurring during tumor regression.

Collagen changes from treated mouse tumors versus vehicle tumors also varied with time. Single-fiber collagen straightness trended toward a curlier phenotype on day 12 of treatment. Similarly, single-fiber collagen length was statistically significant on day 12 of treatment. Interestingly, single-fiber collagen width changed the fastest, with the largest differences observed on day 6 of treatment. This may suggest that collagen straightness and length alterations are more linked to tumor regression or changes in tumor cell signaling, as they changed most at the end of treatment when tumors shrink in response to therapy.^35^ These late collagen straightness and length changes may also reflect immune infiltrate changes in the tumor as treated mice have received the full regimen of *α*-CTLA-4 and IC by day 9, activating T and NK cells while reducing T regulatory cells.^13,14^ By contrast, perhaps collagen width alterations that are most different on day 6 of treatment are more linked to the radiation therapy that occurred on day 0. Radiation therapy can affect fibroblasts within the tissue, leading to overactivation and some-times increased collagen synthesis.^67^ We showed that treated tumor *α*-SMA+ fibroblast numbers increased significantly from days 0 to 6, and collagen density significantly increased at day 6, possibly reflecting the overactivation effect of radiation therapy on the fibroblasts. However, we also showed a trend toward decreased treated tumor *α*-SMA+ fibroblast numbers from days 6 to 9. This day 9 reduction in α-SMA+ fibroblasts may be partly why we see collagen fiber straightness and length change on day 12, as well as indicate reduced collagen deposition^68^ near the end of treatment, though additional assays are needed to confirm this. In a cohort of male head and neck cancer patients, 20 Gy of radiation therapy led to a decrease in collagen III synthesis.^67^ Interestingly, in a cohort of female breast cancer patients who received 50 Gy, the opposite effect was seen following radiation therapy where an increase in collagen I and III synthesis was observed.^69^ We presume the collagen imaged within these melanoma tumors is primarily collagen I with some collagen III contributions.^70^ Additional studies are needed to determine whether collagen synthesis is increased or decreased in our melanoma model; however, our results may follow the trend of the head and neck cancer patients due to tissue similarity, where radiation therapy reduced collagen synthesis. The collagen morphology changes we observed may also be linked to the reprogramming of cancer-associated macrophages and fibroblasts, which have been implicated in collagen deposition and pro-tumor behavior.^20,42,71-76^ Others have shown collagen SHG morphology changes with chemotherapy and targeted therapy, but to our knowledge, this is the first study to quantify these changes during combination radiation and immunotherapy *in vivo*.^43^ As immunotherapy is increasingly common for melanoma and other cancers, it may be clinically important to monitor other biomarkers of response.^12,77^ This work suggests that collagen morphology changes may be prognostic during immunotherapy.

Overall, we found that the CT-FIRE output features of straightness, length, and width were more sensitive to collagen changes with this model and treatment, at both FOV and single-fiber levels, compared with CurveAlign coefficient of alignment and density. This may be because CTFIRE considers single collagen fibers through independent curvelet calculations compared with CurveAlign that calculates collagen changes at a FOV level only where curvelets are dependent on the entire FOV. This was especially true as our dataset did not contain tumor borders, which are needed for the relevant coefficient of alignment calculations.

It is important to note that although nearly all of the SHG signal we gathered is expected to be collagen, there is a chance that other ECM proteins were captured such as elastin.^17,78^ We do not expect that the small contribution of elastin to our SHG signal significantly impacts our conclusions. We also acknowledge that these images were acquired with linear polarization, and since SHG is polarization-sensitive, all collagen fibers may not be excited equally here. However, as all SHG images were collected under the same imaging condition, we do not anticipate this to significantly impact our conclusions.

Although *in vivo* SHG imaging provides information on collagen dynamics across treatment groups and treatment time courses, SHG imaging alone is not sufficient to specify a biological mechanism for collagen changes. Parallel measurements such as flow cytometry, western blot, and single-cell RNA sequencing, some of which we are currently pursuing, should identify specific cells, cytokines, and proteins that drive this reorganization. We are also interested in which component or components of the triple therapy prompted these collagen changes. Future studies may include similar collagen SHG analysis in mice that received only a single component of the therapeutic regimen, as well as expansion of this work to a mouse colon carcinoma model for comparison. Ultimately, this imaging approach could provide insight into tumor microenvironmental changes and collagen dynamics, with key implications in improving immunotherapy response in cancer. This work could also lead to the exploration of collagen biomarkers for additional cancer types.

## 5 Conclusion

Here, collagen morphology features were quantified from *in vivo* SHG images of mouse melanoma tumors during radiation and immunotherapy. Collagen dynamics and phenotypic changes with temporal context were quantitatively examined with CurveAlign and CT-FIRE software to evaluate collagen changes at FOV and single-fiber levels. Collagen from radiation- and immunotherapy-treated mice reorganized during treatment toward a healthier tissue phenotype including: shorter, wider, curlier collagen fibers, with modestly higher collagen density. Temporally, collagen fiber straightness and length changed late in treatment (days 9 and 12), while width and density changed early in treatment (day 6) compared with vehicle-treated mice. Overall, we have shown substantial quantitative and qualitative changes in collagen during the melanoma response to this radio-immunotherapy regimen. SHG imaging in preclinical models or patient samples may provide insight into tumor microenvironmental features that are associated with improved immunotherapy response in cancer, and new biomarkers of response.

## Supplementary Material

supplemental content

## Statement of Discovery

This work uses second harmonic generation imaging to characterize *in vivo* collagen remodeling in melanoma tumors during administration of radiation therapy and immunotherapy. Quantitative collagen changes may provide insight into tumor microenvironmental features that are associated with improved immunotherapy response in cancer, and new biomarkers of response.
