# Supplementary material for: Quantifying in vivo collagen reorganization during immunotherapy in murine melanoma with second harmonic generation imaging
Source: Biophotonics Discov. 2024 May 20;1(1):015004. doi: 10.1117/1.BIOS.1.1.015004 (PMC11247620; doi:10.1117/1.BIOS.1.1.015004)
Supplement: Supplementary file 1 [file BIOS_001_015004_SD001.pdf]

Supplementary Material

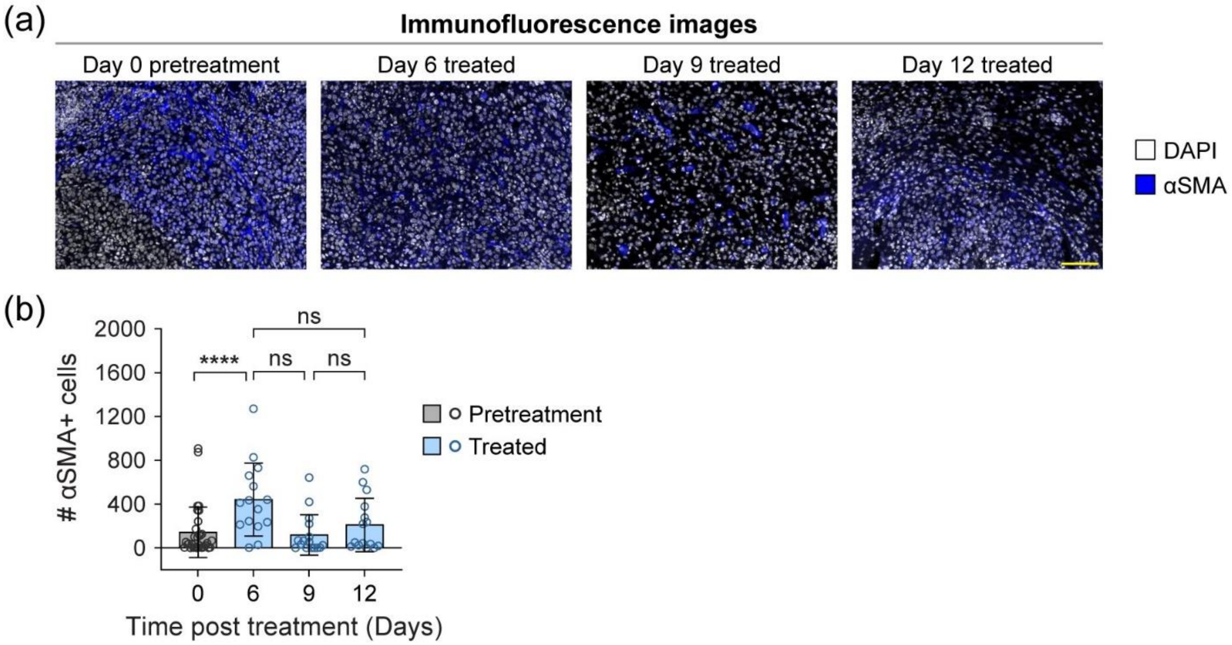

Figure S1: Immunofluorescence of αSMA+ fibroblast populations in mouse melanoma tumors during treatment. (a) Representative 20× immunofluorescence images in B78 mouse melanoma tumors from Day 0 pretreatment mice and Day 6, 9, 12 treated mice (DAPI gray, αSMA+ fibroblasts blue). **(b)** Number of fibroblasts within melanoma tumors from pretreatment and treated mice over time (mean number of fibroblasts Day 0: 143, Day 6: 441, Day 9: 119, Day 12: 210). Bars: mean ± SD, each point represents a FOV. n = 2-4 mice per treatment group, pretreatment Day 0 images n = 32 FOV, treated Day 6 images n = 15 FOV, treated Day 9 images = 16 FOV, treated Day 12 images = 15 FOV. ANOVA with Tukey's HSD, ****$p<0.0001$. Field of view, FOV. Scale bar is 100 µm.

Table S1: B78 mouse melanoma tumor measurements. Tumor volumes from imaged mice, across two experimental replicates. Tumor volumes measured with calipers and reported as mm^3. Shaded cells intentionally blank as mouse was already imaged and euthanized prior to this time point. Pre = pretreatment, V = vehicle, T = treated.

Time Post Treatment (Days)	625891 Day 0 Pre	628497 Day 0 Pre	618134 Day 6 V	610909 Day 6 T	624801 Day 9 V	625892 Day 9 T	610914 Day 12 V	509047 Day 12 T
-30	0	0	0	0	0	0	0	0
-23	0	25.6	41.6	18.0	4.0	4.0	6.0	4.0
-16	87.3	56.5	101.3	56.1	44.0	54.7	61.8	27.9
-9	156.6	58.8	129.6	59.3	103.9	124.9	86.0	37.9
-2	215.0	191.6	207.1	133.1	223.8	234.2	191.7	56.1
5			226.9	268.4	385.8	282.4	228.1	102.7
12							302.4	87.7

Time Post Treatment (Days)	688368 Day 0 Pre	671142 Day 0 Pre	683435 Day 6 V	681652 Day 6 T	685385 Day 9 V	674216 Day 9 T	678977 Day 12 V	668823 Day 12 T
-26	0	0	0	0	0	0	0	0
-19	14.1	26.7	50.8	21.4	0	43.2	46.0	26.1
-12	0	133.4	36.8	83.5	41.2	96.2	71.2	39.7
-8	48.6	63.9	134.8	133.8	72.8	202.2	113.2	45.4
-2	167.1	254.9	132.5	147.3	114.9	219.0	125.3	142.7
2			181.9	398.5	131.4	318.0	174.6	245.0
5			262.0	338.1	228.9	446.2	146.2	327.7
9							297.6	316.5
